# Supplementary material for: Are carbon emissions trading and green financial instruments synergistic? -Comprehensive quantitative research based on content analysis
Source: PLoS One. 2024 Mar 7;19(3):e0298601. doi: 10.1371/journal.pone.0298601 (PMC10919601; doi:10.1371/journal.pone.0298601)
Supplement: S1 Data — (ZIP) [file pone.0298601.s001.zip › Data/地级市Z_F工作报告.zip!/╣π╢1⁄2/╒┐╜¡╩╨/└┤╫╘╣1⁄2╓┌║┼í╛┬φ┐╦╩2╛▌═°í┐_2013.docx]

各位代表：

　　我代表市人民政府向大会报告工作，请予审议，并请市政协委员和其他列席的同志提出意见。

　　2012年工作回顾

　　2012年是本届政府履职的开局之年。一年来，在市委的正确领导下，在市人大及其常委会和市政协的监督支持下，市政府认真落实市第十次党代会和市委十届三次全会的决策部署，坚持好为标准、快字当先、实最关键、绩见高低，不回避矛盾、不避重就轻、不坐而论道，攻坚克难，团结实干，较好完成了市第十三届人大一次会议确定的各项任务，实现经济社会平稳发展。预计全市完成生产总值1900亿元、增长10%，人均生产总值26782元、增长9%；工业总产值2200亿元、增长12%；固定资产投资完成572.3亿元、增长16.6%；社会消费品零售总额900亿元、增长12.1%；地方公共财政预算收入92亿元、增长15%，来源于湛江的财政总收入542.38亿元、增长17.1%；城镇居民人均可支配收入20222元、增长15%，农民人均纯收入9637元、增长16.7%。

　　各位代表！这一年，湛江人民期望多年的宝钢湛江钢铁基地项目获得国家核准，湛江现代工业迎来了发展的春天；这一年，我们顺应群众呼声，全面完成港湾清障，湛江湾重归自然、再现美丽；这一年，我们万众一心，成功抗击13号强台风“启德”的正面袭击，实现人员零伤亡目标，为应对极端灾害天气积累了宝贵经验；这一年，我们组织开展了声势浩大的创卫创模活动，全面完成38个农贸市场升级改造，市场面貌焕然一新，城市环境明显改善；这一年，我们众志成城，完成了北桥河综合整治，河岸鲜花盛开，河水清澈见底；这一年，雷州半岛环岛一级公路列为国防交通重点建设项目，徐闻段正式开工建设，“三环四通”大交通建设翻开崭新一页；这一年，我们建成扶贫开发项目1404个，完成农村困难群众住房改造1.68万户，较好完成了扶贫开发“双到”三年目标任务；这一年，我们建成并免费开放金沙湾海滨浴场，举行了首次海湾横渡，开启了滨海旅游新篇章；这一年，我们精心举办北京、上海、海口三场投资说明会，全面展示了开放的湛江、奋进的湛江、生态的湛江！

　　一年来，围绕推动五年崛起、建设幸福湛江，我们主要抓了六项关系全局的重点工作：

　　扎实推进现代产业体系建设。着力转方式、调结构，大力发展海洋经济，构建现代产业体系。全市三次产业结构由20.2∶41.8∶38调整为19.7∶42.1∶38.2。钢铁基地项目厂东区首期工程动工，自备电厂和30万吨级散货码头完成投资8.7亿元，占投资额24%。中科炼化项目取得用海使用权证书，一期场地平整完成60%。石化园区10.9平方公里围填海工程开工。晨鸣林浆纸一体化项目一期达产达效。渤海菜籽综合加工、东兴炼油200万吨柴油技改等7个项目竣工投产，冠豪高新特种纸基地一期完成投资19亿元，占投资额60%。奋勇经济区完成农场改制，首批8个项目动工建设。服务业加快发展，港口货物吞吐量1.7亿吨、增长10%，集装箱吞吐量41.2万标箱、增长8.3%；中石化国际事业华南总部落户，北京王府井百货进驻开业，霞山水产品批发市场和南方国际水产交易中心两个保税仓建成使用，海田国际车城、建材城和麻章商贸物流城动工；成功竞得省滨海旅游产业园3亿元扶持资金，启动“五岛一湾”滨海旅游开发，完成旅游投资45亿元，增长1.2倍，建成南海明珠游艇码头等项目，旅游业接待游客2239万人次、总收入127亿元，分别增长23.6%和36.7%。

　　扎实推进县域经济和区域协调发展。坚持城乡统筹，推动县域经济提速增效，加快发展现代农业。扎实开展“三讲三评”，县域经济发展掀起新高潮，县（市、区）初步实现月月有签约，县县有项目，年年有投产。全市招商签约项目167个，总投资1143亿元，其中动工63个，建成32个，雷州大唐火电项目获得国家发改委“路条”。5县（市）经济总量全部突破百亿元大关。农业总产值608亿元、增长6.5%，其中现代农业增加值比重达75%。廉江、雷州分别评为全国粮食生产先进县（市），徐闻、遂溪分别评为全省水果、水产品生产先进县。正大集团首期4个项目竣工投产，成为“中国—东盟现代农业合作示范区”；南国花卉科技园开业，入园企业146家；中国对虾湛江价格指数首次在广东价格网公布。建成硇洲、乌石国家级中心渔港主体工程和廉江水库移民“双转移”培训就业基地。农业产业化步伐加快，84家农业龙头企业带动55万农户，户均增收近5000元；农业类广东省名牌产品数量继续居全省地级市第一。

　　扎实推进基础设施和城市建设。全力实施“三环四通”大交通工程，推进重点项目建设，重点项目完成投资260亿元。鉴江供水枢纽工程完成闸坝一期，2.75公里跨湛江湾海底盾构隧道贯通；建成省道廉坡线和500千伏港城变电站扩建等项目，省道塘企线和蓬吴线动工改造。《湛江市城市总体规划纲要（2011-2020年）》通过省部联合审查。完成“三旧”改造项目24个、面积525.7亩。市政重点项目建设完成投资5.2亿元，建成奋勇大道、疏港大道二期和东柳路，完成海北路、绿塘路和12个交通拥堵路口改造，完成赤坎江、寸金渠整治。建成滨湖公园，建设城市绿道15.8公里、生态景观林带163.5公里，造林20万亩，森林覆盖率29.1%，荣获“广东省林业生态市”称号。君豪酒店获鲁班奖，“湛江园”项目获第八届中国（重庆）国际园林博览会金奖。新增省级生态乡镇2个、生态村1个；创建省市卫生村144个，麻章镇通过省卫生镇验收；市区新建改造公厕154座、垃圾中转站35座；城市污水处理率达到90.61%，回收生活垃圾无害化处理率100%；饮用水源水质达标率100%。市区环境空气质量在全国环保重点城市保持前列。

　　扎实推进改革创新和对外开放。医保“湛江模式”在全省推广，城乡居民参保率98.7%，住院报销年度最高支付限额达到25万元；开展公办基层医疗卫生机构综合改革。深化财政管理体制改革，实现财政账户统一管理。探索融资新模式，推动交投集团与黑龙江龙建集团合作，采用“IPC”模式实施“两路一桥”项目。金融改革创新综合试验区建设取得新成效，汇丰、东亚、招商银行落户湛江，平安、建信人寿和华泰财险等证券、保险公司在湛设立机构；湛江商行成功更名广东南粤银行；金融机构存贷比达56.2%。推进资源价格改革，实施阶梯式电价。外经贸持续发展，外贸进出口总额46.9亿美元，其中出口22.1亿美元，增长5.4%；实际利用外资8726万美元，增长64%。成功举办首届中国—东盟企业合作交流年会和第4届亚太批发市场大会。与美国大西洋城、休斯敦缔结友好城市。建设开明政府，举行9场市长工作午餐会和10场市政府新闻发布会，大力推行网络问政，市政府领导全部轮流与网民互动交流。

　　扎实推进民生和社会事业发展。市财政民生投入157亿元，增长16.5%，占地方公共财政支出73%。18件民生实事全面落实。启动教育创强工程，赤坎、霞山两区和26个镇（街）通过省督导验收。高中阶段教育毛入学率89%；职教基地首期项目动工，市四中新校区基本完工，建成规范化中小学校501所。完成607家农家书屋配套，市直3家文艺团体和两家电影公司实现转企改制；举办“雷州历史文化展”和湛江海洋图书文化节。专利申请量、授权量增长10%，实施产学研合作项目206个。城乡居民养老保险基本实现全覆盖，2.17万名困难国有集体企业退休人员纳入政府资助基本医疗保险。动工建设中心人民医院新院，完成市120紧急医疗救援指挥中心选址前期工作，行政村卫生站覆盖率100％。省运会主场馆完成投资8亿元，占工程总量35%；建成跳水运动学校；湛江籍运动员刘鸥、陈晓君、何冲参加伦敦奥运会，取得1银两铜好成绩。市档案馆新馆主体工程封顶。二轮修志编纂基本完成。强化食品药品监管，在全国率先建成301家“阳光厨房”。人口计生工作评为全省先进。第四次荣获“全国双拥模范城”称号。人防、统计、粮食、防震减灾、气象预报、民族宗教、妇女儿童、老区建设等工作取得新成效，海关、检验检疫、海事、边检等口岸单位为我市扩大开放作出新贡献。

　　扎实推进民主法制建设。人大建议、政协提案办复率100%，评为全省建议提案承办工作先进单位。开通省网上办事大厅湛江分厅，清理废止过时规章制度538件。行政复议案件结案率100%。深入开展“三打两建”，市场秩序、投资环境进一步优化。领导干部接访和下访制度化、常态化。加强综治信访维稳平台建设，完善应急管理体系；组织“南粤亮剑012战役”，严重暴力和涉枪案件下降，社会治安持续好转；建成海防与打击走私监控指挥中心；落实安全生产“一岗双责”，有效保障人民群众生命财产安全。

　　各位代表，过去一年，在全球经济低迷不振、国内经济下行压力加大的背景下，我们能够取得上述成绩极为不易，这是中共湛江市委正确领导、科学决策的结果，是市人大、市政协有效监督、大力支持的结果。成绩归功于各级人大代表和政协委员的共同努力，归功于全市人民的团结奋斗，归功于驻湛部队、驻湛单位和海内外朋友的鼎力支持，湛江市人民政府感谢你们！并向你们致以崇高的敬意！

　　回顾一年工作，我们清醒认识到，我市经济社会发展还存在一些问题：钢铁基地项目主体工程未按计划动工，中科炼化项目征地拆迁未按计划完成；海湾大桥西连接线等重点项目未按计划竣工；年初确定的部分经济增长目标未能如期实现；南方大港地位受到挑战；民生保障能力与人民群众的期待仍有较大差距，社会管理和公共服务体系有待完善，等等。对此，我们将深刻剖析，认真解决。

　　2013年工作安排

　　新的一年，我们要深入学习和贯彻落实党的十八大精神，坚持以邓小平理论、“三个代表”重要思想和科学发展观为指导，深化改革开放，优化发展环境，强化招商引资，扩大有效需求，提高创新能力，保障改善民生，加快推进工业化、城镇化、国际化、生态化和农业现代化，全力以赴做大做优经济“蛋糕”，苦干实干开创大工业时代，努力保持经济平稳较快发展，为尽快达到全国发展平均水平，推动五年崛起奠定坚实基础。

　　今年我市经济社会发展的预期目标是：生产总值增长12%，人均生产总值增长10.5%；固定资产投资增长20%以上；工业总产值和增加值分别增长18%、15%；港口货物吞吐量增长10%；社会消费品零售总额增长15%；居民消费价格指数涨幅控制在3.5%左右；外贸进出口总额增长5%，实际利用外资增长10%；地方公共财政预算收入增长15%，人均公共财政收入增长13.5%；城镇居民人均可支配收入增长11%，农民人均纯收入增长12%；城镇登记失业率控制在3.5%以内；人口自然增长率控制在8.3‰以内；万元生产总值能耗下降3.66%；市区生活污水处理率94%；完成省下达的节能减排约束性指标。实现上述目标，必须着力抓好以下工作：

　　一、壮大主导产业，努力推进工业化

　　强化工业主导地位。湛江五年崛起，成败在工业。坚持以钢铁、炼化项目建设为契机，充分发挥港口优势，充分利用国内外两种资源、两个市场，加快发展临海现代工业，着力把工业总量做大、结构做优、实力做强。今年重点建设项目84项，总投资2929亿元，年内完成投资321亿元。推动湛江钢铁基地项目按计划3月启动设备上网招标，三季度开工建设钢铁主体工程，年内完成投资52亿元。推动中科炼化项目按计划上半年完成场地平整，启动海域疏浚和重件码头配套设施建设，下半年动工建设炼油主体工程，年内完成投资40亿元。开工建设晨鸣林浆纸一体化项目二期，完成投资4亿元。建成吴川威立电线电缆、30万吨调合润滑油、华海结晶果糖和航运集团船厂4万吨级船坞等项目。完成华德力电气项目主体工程，动工建设雷州大唐火电、一品家具产业基地、中能酒精燃料乙醇和廉江华润新型水泥项目，5个项目完成投资29.2亿元。

　　着力培育三大经济增长极。全力推动东海岛、奋勇经济区、南三岛由三大发展平台向三大经济增长极转变。东海岛既是开创大工业时代的主战场，也是五年崛起的第一增长极，经济技术开发区作为开发建设的责任主体，要进一步保持势头、提升劲头、当好龙头，不负众望，真抓实干，始终以如期建成钢铁、炼化项目为己任，以主人翁姿态当好“服务员”、做好“协调员”、干好“办事员”；以建设东海岛工业新城为使命，统筹谋划中南部临海区域，规划建设生活、行政、商务、教育卫生和公共服务五大中心。基础设施重点动工南部围填海工程，建成东海岛疏港公路，开工建设岛东大道、岛南大道等9条主干道和500千伏输变电工程，完成东海岛至雷州高速公路前期工作，建成东简首期3万吨污水处理厂。产业发展重点动工建设中海油80万吨重交沥青、广东鹏尊能源、京信热电联产和投资150亿元的中国纸业东海岛高端纸种等项目；加快申港钢铁配套加工中心等6个在建项目建设；建成冠豪高新特种纸基地一期和双林医药产业园等项目，完成石化园区10.9平方公里填海首期工程；规划建设并申报设立西南部10平方公里国家级高新园区；整合提升龙海天省级滨海旅游度假区。奋勇经济区要编制完成发展规划和总体规划，建立土地储备管理分中心，重点推进8个入园项目建设，开工建设国家级医疗器械检验中心。南三岛要按照打造国家级滨海旅游示范区和中国南方冬休度假基地的目标，创新管理体制，编制完成《南三岛分区规划》和旅游发展规划。加快岛内基础设施建设，扩建18.6公里贯岛公路。

　　提升现代服务业发展水平。大力发展港口物流业。湛江港集团作为实施港口带动工程的龙头，要不辱使命、主动作为，加快港口发展战略转型，走出市区、带动半岛、开拓腹地、开放合作，大力发展临港产业，大力提升港口服务和对外合作水平，大力改善港口发展条件，再创南方大港新优势。加大港口建设投资力度，建成宝满集装箱码头一期，启动二期工程，动工建设东海岛新港区。加强港口工作领导，出台鼓励集装箱业务发展的政策措施。完善海安港管理体制，建设徐闻新港，开工建设南山客滚码头，打造琼州海峡运输枢纽，对接海南国际旅游岛。大力发展旅游业。用好用活省滨海旅游扶持资金，支持旅游投资集团整合资源，加大产业招商，推进“五岛一湾”滨海旅游开发；加快实施吉兆湾国际海洋生态度假中心首期等18个重点旅游项目；大力推介湛江旅游形象标识，推广“湛蓝的海、湛蓝的天”旅游口号，不断提高湛江知名度、美誉度。举办首届“湛江海洋周”。着力打造旅游精品，重点抓好“八个一”：建成一条海鲜美食街、一条特色风情街，推出“五岛一湾”一日游、市区人文一日游和雷州半岛一日自驾游线路，开发一系列特色旅游文化商品，打造一台特色民俗文化节目，保护修复一批近代建筑物。大力发展商贸服务业。推进金融改革创新综合试验区建设，推动光大、兴业等银行落户，打造人民大道中金融服务一条街；建成一批高档写字楼和大型超市；支持平铝集团五星级酒店、红星国际广场、赤坎东盟城等项目落地开工；加快建设中央直属储备糖库等大型商贸项目；支持本市地产企业做大做强，引入更多有理想、有文化、有实力、有信誉、有品牌的“五有”地产企业进入湛江，参与城市建设，提升城市品质。大力发展智慧生活服务业。落实与中国电信、中国移动、中国联通广东省分公司签署的战略合作框架协议，积极引入市场资源，拓展电子商务，发展智能交通、智能家居、智能楼宇，建设智慧湛江。

　　深入开展“三讲三评”推动县域经济发展。进一步丰富讲评内容，科学设置考核指标，加大财政支持力度，鼓励以先进理念和科学规划引领园区发展。支持佛山顺德（廉江）、深圳龙岗（吴川）省级产业转移工业园区扩园发展，年内廉江园区引进项目12个、投资25亿元，建成5个项目、动工4个项目、完成投资10亿元；吴川园区引进项目15个、投资22亿元，建成8个项目、完成投资5.2亿元。支持坡头科技产业园、遂溪岭北工业园和太平森工产业园申报省级产业转移园。

　　加大招商融资力度。落实基投、交投、水务和旅游投资集团项目资本金，推进融资平台与土地储备中心有效整合，支持参与土地一级开发。充分发挥六大专业招商分局作用，全面提升招商引资质量和实效。继续大力发展民营经济，支持中小企业健康发展，鼓励全民创业。坚持信守承诺，主动服务，以更强的力度实施“十百千”投资计划和“乡贤反哺工程”，吸引更多企业家感情回乡、资金回流、企业回迁，支持广大乡贤回归本土、扎根桑梓、建设家乡！湛江发展的潜力在民资、动力在民企、活力在民营，我们要满怀激情招商、饱含真情安商、富有温情亲商，让广大民营企业家更有地位、更有荣誉、更有奔头！

　　健全服务企业机制。完善百家重点企业“直通车”制度，搭建政府服务企业的便捷通道；加强面向企业的规划、投资、用地、环保、融资信息服务，建设服务企业公共技术平台。落实支持企业转型升级优惠政策，进一步清理规范涉企收费事项，除法律规定和上级统一部署外，市政府不再出台新的收费项目，真正为企业营造宽松的发展环境。

　　二、夯实“三农”基础，努力推进农业现代化

　　加快正大项目建设。推进正大(湛江)现代农业综合开发基地项目建设，年内完成投资20亿元。重点建设遂溪岭北饲料厂、洋青肉鸡食品加工厂、城月孵化厂和两个大型超市；建成遂溪乐民种鸡场、洋青肉鸡场、雷州客路种猪场和种猪配套场。

　　推进农村土地流转和规模经营。完善土地承包关系，创新土地流转模式，健全土地流转市场，规范土地流转管理，鼓励农民依法、自愿流转土地承包经营权，发展土地适度规模经营。建立县级农村土地承包经营纠纷调解仲裁机构，开展县（市）镇两级调解仲裁员培训。

　　发挥农垦示范带动作用。深化垦地互动合作，组织编制湖光农场控制性详细规划，合作开发湖光农场片区，打造海峡两岸农业合作试验区中心区、现代农业休闲观光园区和湛江城市新组团。支持农垦新场居建设。推进徐闻海鸥旅游观光农业综合示范项目，建成徐闻南华农场休闲度假农庄。加快农垦国家级现代农业示范区建设，建成广前糖业原糖加工和农业部万亩甘蔗机械化示范基地等8个项目。

　　改善农业生产条件。修编土地利用总体规划。加快鉴江供水枢纽工程建设，上半年具备供水条件；完成13宗重点小型病险水库除险加固工程，开工建设麻章临海大堤等4宗海堤加固达标项目。建成小型农田水利工程7个示范镇、3个省级重点县。建设现代标准农田75万亩。建成农海产品检测中心。投入2.38亿元改造农村电网。促进信息技术与现代农业有机融合。实施海洋气象灾害防御保障工程，提升气象现代化水平。

　　完善农产品流通体系。实施“双百市场工程”，加快建设广东北部湾农产品流通综合示范园区，升级改造徐闻、廉江横山两个农产品交易市场，打造面向全国和东盟的大型农产品集散地。

　　三、加快扩容提质，努力推进城镇化

　　加快城市新区建设。新区是抓手，新区是亮点，新区是希望。要以海东新区、中央商务区、城市原点广场和西城新区建设为重点，加快做大做强做畅做美中心城市，进一步增强集聚、辐射和带动能力，成为区域经济发展的龙头和引擎。海东新区建设要以省运会主场馆片区为重点，完成征地1680亩，搬迁三柏西等3条村庄，建设搬迁农民公寓；动工建设海东新区岸线和滩涂综合治理工程，完成拆迁补偿和海域使用申报，完成吹沙量1000万立方米；建设中科炼化职工生活区，开工建设海东大道、东海岸观海长廊等7条主干道，打造海湾城市新名片。中央商务区建设要加快龙潮东路等14条7.2公里市政道路建设；加快平乐工业区企业外迁，改造龙潮整村和平乐下村365亩旧村场，推进城市空间置换，集聚发展高端服务业，打造海湾城市新亮点。城市原点广场建设要加快湛江港集团霞山旧港区功能退出，上半年完成规划报批，三季度完成首期项目用地拆迁，优选引入投资主体，打造海湾城市新地标。西城新区建设要完成分区规划编制；开工建设湖光国际商贸中心；推进“大湖光岩”休闲旅游圈和湖光交椅岭生态整治，建成开放湖光郊野公园；升级改造湛江西站，动工建设湖光客运站，实施疏港大道改市政道路工程，打造海湾城市新片区。

　　提升城市发展承载力。加大力度实施“三环四通”工程，完善现代交通体系，密切与珠三角和周边城市的联系。上半年完成广湛高速官渡出入口立交改造，年内建成海湾大桥连接线二期和省道蓬吴线改建项目；雷州半岛环岛一级公路徐闻段完成投资10亿元，开工建设雷州环城东段；开展霞山至南三岛、调顺岛至海东新区海底隧道和城市轨道交通项目前期工作；建成茂湛铁路，积极推动西部沿海高铁江茂段开工建设；动工建设东海岛铁路。加快湛江机场迁建前期工作，确定新机场选址及投融资模式。完善城市道路体系，重点改扩建市政道路20条，上半年完成海滨大道改扩建；建成新湖大道、社坛路；改造椹川大道、康顺路、康宁路、跃进路、中山一路和机场路；开工建设高速公路连接中心城区的湛江大道；开工建设广州湾大道，连接赤坎、霞山观海长廊，形成亮丽的观海风景线。完成一批中心城区小街小巷硬底化改造。鼓励更多社会力量参与繁华地段地下隧道等市政建设。注重提高城市规划和重要建筑物、建筑群设计水平，打造优美的城市天际线。实施海湾西岸和海湾大桥光亮工程，扮靓璀璨夜景湛江湾。扩建金沙湾广场，改造南珠广场，提升时代广场，与新建的城市原点广场形成城市广场群，成为滨海旅游新景点。

　　推动城乡发展一体化。坚持以城带乡，统筹城乡发展，加快城镇化进程，打造支撑国家开发南海战略的大后方。完成湛江城乡发展战略规划编制。坚持以县（市）城区为核心、以中心镇为主轴、以中心村为基础，建设一批卫星城镇和农村新社区，建成一批省级名镇、名村和市级示范村。以实现城乡公共服务均等化为取向，推进公交、物流、教育、文化、卫生、科技、广电、信息等公共设施向农村覆盖，建成50条基本公共服务均等化示范村；新增农村客运班车50辆；新建2个县（区）图书馆和达标文化站21个、文化室389个，启动广播电视“户户通”工程；深化户籍制度改革，完善积分制入户城镇政策，有序推进农村转移人口市民化，构建整体协调、服务共享、互动发展的城镇体系。

　　加强和改进城市管理。城市管理重点突出“一清三治”，即清理闲置土地和治水、治堵、治乱。加大力度清理闲置土地。加快清理城市核心区未开工建设项目，重点清理并依法依规分类处理海滨大道、人民大道和椹川大道周边闲置土地。加大力度治“水”。以北桥河成功整治为样板，用铁手腕、硬措施整治南柳河，全面完成截污、清淤、绿化，年内实现岸变绿、景变美、水变清！大力整治赤坎水库，实现水质稳定，环境秀美，形成全长7.8公里的休闲绿道。动工建设南方路、海丰路、海湾路等5条道路地下排水管网。加大力度治“堵”。坚持公交优先发展，改革公交管理体制，实施公交路权优先，规划建设地面大运量快速公交系统，城市公交车万人拥有量达到12标台；完成椹川大道与文明路等5个主要拥堵路口升级改造；建造一批市区停车场，增设停车位5000个；建成交警智能指挥中心主体工程，完善交通灯控设施，分时分段有效疏导交通；坚决取缔机动车非法营运，规范电动车管理，健全公共自行车道路网络。加大力度治“乱”。建设数字化城市管理系统，落实网络化管理责任。全力整治市区“六乱”，强化市容卫生和环境秩序管理考核；规范街区广告和标语张贴悬挂；加强泥头车、建筑垃圾和防尘管理。严厉打击违法建设行为。

　　四、优化开放环境，努力推进国际化

　　建设法治化国际化营商环境。继续改善政务环境，做到高效透明。加强法治环境建设，规范行政裁量权和执法行为、执法程序，完善政府公共服务制度规章，推广商事网上登记注册，简化投资管理流程，推行项目立项阶段用地预审、规划选址、环境评价等手续同步并联办理，实现企业投资便利化。着力促进人情社会向法治社会转变，从领导做起，从科长做起，从科员做起，让企业和群众不请客、不找熟人、不托关系就能快办事、好办事、办成事！继续改善诚信环境，做到信用共守。加快建立银行、企业、公职人员、普通公民诚信体系，将工程中标企业分包行为纳入不良信用记录，强化信用信息在政府采购、项目承包、融资信贷、工程招投标等领域的运用，着力打造“诚信湛江”。继续改善治安环境，做到安全稳定。实行公安经费财政保障，支持公安依法高效履职，杜绝以罚代管、只罚不管现象；深入推进“三打两建”，重点铲除保护伞，斩掉利益链；加强农村治安和维稳工作，建设廉洁村镇；进一步净化发展环境，让投资者放胆投资、放手创业、放心发展！

　　促进对外贸易稳定增长。坚持进出口并重，保持外经贸平衡发展。推动出口商品技术、品牌、质量、服务创新，建设国家级水海产品、省级小家电外贸转型升级示范基地，提升“湛江对虾”、“湛江电饭锅”等区域品牌影响力。完善口岸基础设施，推行“一次申报、一次查验、一次放行”和24小时预约通关模式。支持企业“走出去”开展跨国经营。争取宝满保税物流中心（B型）获批，扩大海港空港开放，加快东海岛综合保税区申报工作。

　　提高开放合作水平。加强海外资源合作，建立新型海外资源供应基地。强化粤西区域合作，推进粤西组合港建设，共建“湛茂阳”临港经济圈。主动参与珠三角、北部湾经济区和海南国际旅游岛经济合作，积极融入粤港澳经济圈。扩大与台湾工商团体、企业交流合作。探索建设中国东盟产业合作示范区，办好中国（广东）—东盟合作华商交流会。提升国际友城工作水平。

　　五、坚持绿色发展，努力推进生态化

　　湛蓝的海、湛蓝的天，是大自然赋予湛江人民的厚礼，我们必须坚守“既要经济崛起，又要蓝天碧水”的底线，努力建设天蓝、地绿、水净的美好家园，让湛江永远美在生态自然，美在和谐共生，美在永续发展！

　　推进资源能源节约。转变资源利用方式，坚持环境资源谁开发、谁治理，谁收益、谁补偿，促进环境资源合理利用和保护。完善《投资项目用地管理办法》，健全工业、建设用地使用权招拍挂出让制度，严格规范建设用地最低投资强度、容积率和主体建筑高度，促进节约集约用地。厉行节能，今年起新建大型公共建筑强制实施绿色建筑标准，大力推广LED照明应用。实施重点领域节水、节电工程，完善居民阶梯水价电价，推广节水灌溉技术。实施一批制糖、风力、太阳能、压差发电和水资源再生利用示范项目，促进资源能源减量化和再利用。

　　强力抓好创卫创模工作。落实“五城同创”目标，重点抓好“创卫”迎检，加强河涌整治、灭蚊达标和健康教育工作，综合整治城市出入口、城郊结合部、城中村和小街小巷脏乱差，强化“六小”行业、农贸市场、宾馆酒店、车站码头等窗口行业监管，年内实现创建国家卫生城市目标，创建国家环保模范城市工作取得重大进展。

　　开展生态文明创建活动。践行尊重自然、顺应自然、保护自然的理念，全面落实创建生态文明区城镇村五年行动计划。年内建成生态文明区1个、生态文明城1个、生态文明镇13个、生态文明村1780条。创建幸福村居，整治农村环境，推广“户收集—村集中—镇转运—县（市）处理”农村生活垃圾收运处理模式，每个自然村建成一个以上生活垃圾收集点；推进“万村绿”行动，打造生态美、村容美、庭院美、生活美、乡风美的“五美”乡村。

　　加强生态环境建设。加快建设雷州、徐闻生态区和雷州半岛绿色生态屏障；编制实施钢铁石化循环经济总体规划，按照国家级循环经济示范区要求，积极推进东海岛循环经济项目与钢铁、炼化主体工程联动建设，做到工业与环保同步推进，城市与产业同步发展，经济增长与环境质量同步提高。加强饮用水源保护，动工建设鹤地水库上游和周边地区生活污水、垃圾处理设施；动工建设合流水库备用水源工程，建设麻章、东海岛自来水厂。以建设“森林城市”为目标，推进林业生态工程和城市增绿工作。建成坡头污水处理厂和市区生活垃圾填埋场三期，动工建设赤坎水质净化厂三期和奋勇经济区、开发区平乐污水处理厂，完成华港工业园及沿线污水治理工程。强化海洋环境综合治理，巩固海湾清障成果，永葆湛江湾白云与海鸥齐飞、碧海共蓝天一色！

　　六、发展社会民生事业，创造幸福美好生活

　　着力发展社会事业。努力办好人民满意的教育，推进学前教育普及化、高中教育优质化、学校建设规范化，实现义务教育均衡发展。重点创建两个教育强县（市、区）、30个教育强镇（街）；启动万名农村中小学教师培训计划；新建12所乡镇中心幼儿园，改扩建14所镇级幼儿园、300所村级幼儿园；创建一批乡土特色文化校园；加快教师公租房、周转房建设。修订实施科技奖励办法，重奖有突出贡献科技人才；坚持市政府和高校合作联席会议制度，推动高校科研课题、学科建设与地方经济社会发展需求紧密融合；推进建设技术创新服务平台和科技孵化器，促进科技成果产业化。动工建设市文化艺术中心，加快粤桂边革命传统教育基地建设；加强文化遗产保护和历史文化街区建设。完成省运会主场馆主体工程，加快分场馆建设，有序推进第14届省运会筹备工作，办好市第12届运动会。全面实施国家免费孕前优生健康检查。加快发展残疾人、妇女儿童、外事侨务、民族宗教、老区建设、史志档案、防震减灾等事业，完善军地联席会议制度，深化双拥共建工作，抓好国防教育和人民防空等工作。

　　着力完善社会保障。努力扩大就业规模，提高就业质量，完善人力资源公共服务平台，新增城镇就业7万人，提供旅游就业岗位3000个；新增农村劳动力转移就业9万人、实现创业2000人。推进社会保险扩面征缴，社会保障卡持卡人数达100万以上。坚持应保尽保，城乡低保补贴标准分别提高到每人每月242元和109元。完善居家养老社会服务，健全社会养老服务体系，引入民资发展养老事业。加快建设广东医学院附属医院新院区，建成市120紧急医疗救援指挥中心；推进县级公立医院改革；完善医保“湛江模式”，提高住院报销比例，医保参保率98%以上；新建20家平价药房；建设区域性医疗医药研发服务中心。

　　着力加强社会管理。坚持民生优先、服务为先、基层在先，完善党委领导、政府负责、社会协调、公众参与、法治保障的社会管理体制。培育发展社会组织，壮大社工、志愿者队伍，支持社会力量承办社区服务机构。推进政府向社会组织转移部分社会管理职能，开展政府购买就业、教育、卫生、住房保障等社会服务。建成使用“12345政府服务热线”平台。健全社会矛盾纠纷调处机制。进一步加强基层反走私综合治理。落实“一岗双责”制度，加强安全生产监督管理。强化药品电子监管，推进“阳光厨房”建设，严厉打击制售伪劣食品药品违法犯罪行为，确保人民群众饮食、用药安全。加强物价调控，确保市场价格基本稳定。积极创建平安湛江，建设应急指挥和突发事件预警信息发布平台，严厉打击涉枪、涉赌、涉毒、涉黑和“两抢一盗”严重刑事犯罪，完善立体化社会治安防控体系，保障人民群众安居乐业。

　　着力办好15件民生实事。完成南柳河综合治理；完成赤坎水库周边环境整治；完成椹川大道东菊苗圃、中山一路南华市场、康顺立交桥3个“水浸街”路段整治；建成三岭山森林公园300亩扩展区，加快生态复绿工程和10公里环园绿道建设；开工建设保障性住房4000套，竣工交付2000套；改造农村低收入困难家庭住房1万户，完成20条贫困村640户整村搬迁，新建水库移民新村85条；动工建设500张床位的市级养老院；新建社会治安视频监控平台27个、视频监控摄像头3万个；落实医疗保险省内异地就医联网住院费用即时结算；加快中心人民医院新院建设，完成土建工程投资50%以上；加快建设职教基地，建成机电学校一期工程；建成两间特色博物馆；建成中心城区公共自行车首期租赁点50个；完成农村公路硬底化改造1000公里；新增“村村通自来水”行政村66条。

　　七、加强政府自身建设，进一步提高政府工作效能

　　打铁还需自身硬。各级政府必须坚持行政为民、勤政立信、廉政树威，加强自身建设，增强自我完善、自我革新和自我提高能力。

　　必须密切与人民的联系。重视完善政府新闻发布会制度，及时发布政府信息，解释政府政策，回应市民关注；完善网民在线互动制度，市政府和部门领导要坚持通过网络与网民加强交流，及时回应网民关注的热点问题；完善市长工作午餐会制度，及时听取社会各界人士对政府工作的意见和建议；完善人民来信来访制度，及时回应群众诉求；认真办理人大代表建议和政协委员提案。充分保障人民群众的监督权、知情权、参与权和民意表达权，直面群众呼声，回应人民关切，建设人民满意政府。

　　必须加深与人民的感情。全体公务员务必心中始终装着群众，放下架子、扑下身子，带着感情联系群众，带着感情落实工作，带着感情破解难题。征地拆迁既是推进项目建设的主要瓶颈，也是事关群众利益的重大问题，只要我们强化项目所在地县（市、区）政府、管委会征地迁拆主体责任，坚持征地拆迁与解决群众困难、增进群众感情结合起来，坚持依法征拆、阳光征拆，真诚善待群众，征拆工作就不会有我们想像的那么难！

　　必须在阳光下为民服务。建成市行政服务中心新办事大厅，建设网上办事大厅二期。今年起，政府各部门要编制并公布行政职权目录，重点公开财政预决算、“三公”经费、保障性住房、食品安全、征地拆迁、价格收费等热点内容和民生信息；所有面向社会提供公共服务的企事业单位，要上网公布服务承诺、服务程序、服务时限、收费标准、监督渠道等内容，让市民看得明白，企业看得清楚，社会看得透亮，让权力在监督的阳光下运行！

　　各位代表，人民是政府的姓、是政府的根、是政府的本。各级政府、全体公务员必须明白：没有人民，哪有人民政府！我们必须时刻把人民利益放在首位，时刻与群众保持血肉联系，时刻从群众中汲取前行的智慧和力量，时刻对人民满怀感谢之意、感激之情、感恩之心，永远不要疏远老百姓，永远不要脱离老百姓，永远不要漠视老百姓！

　　强化督办权威。督办是落实，是效能，是政府执行力。认真落实政府工作督办制度，选派最得力、最熟悉业务、最有责任感的干部加强和充实督办队伍，增强督办权威、提升督办效率，坚持督办事项不解决不放过，牢牢盯紧不落实的事，督促不落实的人，追究不落实的责任，确保政令畅通、有令必行、有行必果！

　　提升素质能力。面对知识不足、能力不足的危险，全体公务员要坚持把学习当作一种政治责任，向书本学习、向实践学习、向群众学习，加快知识更新，努力把学习成果转化为谋划工作的思路、领导工作的本领、推动工作的能力；坚持把“干”作为一种基本能力，作为解决一切矛盾和问题的有效办法，全力营造“干”的氛围，增强“干”的本领，爱护“干”的干部，在“干”中统一认识，在“干”中解决矛盾，在“干”中推动发展。

　　改进工作作风。扎实推进依法行政，完善政府领导出庭应诉和法律顾问制度。完善惩治和预防腐败体系，落实党风廉政建设责任制，严格执行领导干部廉洁从政制度规定，加强审计监督、执法监察和效能监察，确保干部清正、政府清廉、政治清明。大兴求实务实落实之风，践行实干兴市，坚持在监督下实干、在阳光下实干、在摸索中实干。全面转变作风，市政府领导到基层调研一律轻车简从，陪同市直部门不超过3个，县（市、区）陪同领导不超过2人，不贴标语，不搞迎送，不铺地毯，开会不摆花草；简化接待，提倡自助就餐、不上酒水，餐饮时间不超过1小时；精简会议，提倡开1小时的会。坚持勤俭节约、艰苦创业，集中资金改善民生，各级政府要坚持生活过紧日子、工作过苦日子，努力让人民过上好日子！

　　各位代表！实现五年崛起，时不我待；建设幸福湛江，更需努力。今天，我们已经站在新的起点，迈上全面建成小康社会的新征程。时代赋予的机遇，等待我们把握；加快发展的考验，等待我们担当；幸福未来的呼唤，等待我们开创。让我们为之勇往直前，为之攻坚克难，为之团结奋斗，不断夺取振兴湛江新胜利！
